# Supplementary material for: Are carbon emissions trading and green financial instruments synergistic? -Comprehensive quantitative research based on content analysis
Source: PLoS One. 2024 Mar 7;19(3):e0298601. doi: 10.1371/journal.pone.0298601 (PMC10919601; doi:10.1371/journal.pone.0298601)
Supplement: S1 Data — (ZIP) [file pone.0298601.s001.zip › Data/地级市Z_F工作报告.zip!/┴╔─■/┤≤┴1⁄4╩╨/└┤╫╘╣1⁄2╓┌║┼í╛┬φ┐╦╩2╛▌═°í┐_2009.docx]

各位代表：

　　现在，我代表市政府向大会报告工作，请予审议，并请市政协各位委员提出意见。

　　一、2008年政府工作回顾

　　2008年，是我市振兴发展历程中很不平凡的一年。我们分享了北京奥运会成功举办的喜悦，经历了汶川大地震的举国同悲，遭受了世界金融危机的严重冲击。面对复杂多变形势，在党中央、国务院，省委、省政府和市委的正确领导下，市政府以科学发展观统领全局，带领全市人民战胜了各种困难，励精图治、扎实工作，全面完成了十四届人大一次会议确定的各项任务。

　　预计全市实现地区生产总值3858亿元，增长16.5%；万元GDP综合能耗下降4.2%，主要污染物排放总量平均削减3%；完成全社会固定资产投资2510亿元，增长30%；完成地方财政一般预算收入339亿元，增长26.5%；实现社会消费品零售总额1180亿元，增长20%；完成外贸进出口总额448亿美元，增长23.4%；实际使用外资50亿美元，增长58.6%；城镇居民人均可支配收入17500元，增长15.8%；农民人均纯收入9818元，增长17.3%；城镇登记失业率为2.4%。

　　过去的一年，是本届政府的起始之年。我们以改革开放和结构调整为主线，坚持科学发展、和谐发展，一些重点领域的改革取得重大突破，一些事关大连未来发展的重大项目快速推进，一些重大民生问题得到有效解决，大连已步入率先全面振兴的新阶段。

　　（一）国际航运中心建设实现重大突破。大连港与锦州、葫芦岛港签署了战略合作协议，与丹东港的合作正在顺利推进，港口资源整合取得突破性进展。大窑湾保税港区政策功能辐射东北腹地，长春、吉林内陆港正式运营，与沈阳、哈尔滨、满洲里等城市和沈阳铁路局签署战略合作协议。普洛斯-集发沈西物流工业园开工建设。《大连港总体规划》获国家批准，辽宁电子口岸正式开通。

　　重大港航设施建设力度加大。大窑湾集装箱码头17#、18＃泊位建成投产，新30万吨原油码头和中石油LNG码头快速推进，烟大铁路轮渡正式运营。

　　集疏运体系建设加快。土羊高速、沈大与丹大高速连接线、丹大高速延长线正式通车，我市高速公路骨干网络和区域路网基本形成。大窑湾区域铁路网络初具规模，大连铁路集装箱中心站、东北东部铁路岫岩至庄河段开工建设。

　　预计全年完成海港货物吞吐量2.5亿吨，集装箱吞吐量453万标准箱，分别增长10.3%和18.8%。两岸直航大连至台湾航班正式开通。空港新增国际通航点15个，国际航线数量位居全国第四。完成旅客吞吐量822万人次，增长12.8%。

　　（二）新型产业基地形成集聚效应。“两区一带”建设成效显著。重工·起重风电机组扩能改造项目实施，船用曲轴批量生产，中国一重核岛项目建设加快；德国大众30万台自动变速箱项目开工建设，美国博格华纳变速箱模块项目签约；大连湾临海区和大窑湾临港区初步形成了世界级的重装备、汽车零部件产业集群。STX第一条船下水，中远造船、船柴扩建项目快速推进，船舶重工长兴岛造船基地开工建设，渤海岸线造船产业带已经形成。大石化2000万吨炼油扩能竣工投产，逸盛·大化PTA和福佳·大化PX项目开车调试，大孤山半岛石化产业链条形成。全市规模以上工业增加值增长24%。

　　高新技术产业加快发展。进一步拓展了旅顺南路软件产业带发展空间，美国思科等6个世界500强公司的软件项目相继落户，东软国际软件园一期、腾飞软件园二期等项目竣工，天地软件园加快建设，大连（日本）软件园在东京开园，我市获批国家信息产业高技术产业基地。软件和信息服务业销售收入306亿元，增长42.3%；出口10.5亿美元，增长47.4%。英特尔芯片项目快速推进。数控、光电子等高技术产业集群初步形成。全市实现高新技术产业增加值802亿元，增长33.2%。

　　现代服务业呈现良好发展态势。星海湾国际金融中心期货大厦封顶。花旗银行等5家金融机构落户大连，百年人寿保险、渣打银行大连分行获准筹建，大连银行开设北京分行。大商所期货交易额达到27万亿元，增长128.5%。物流业发展势头强劲，日通、近铁等一批世界知名物流企业在保税区、开发区集聚。现代商业发展迅速，引进了瑞典宜家家居等一批现代商业项目。世茂嘉年华等一批旅游大项目建设全面推进，休闲农业和乡村旅游加快发展。全年接待海内外游客3095万人次，旅游总收入400亿元，增长23%。

　　（三）对外开放跃上新台阶。沿海经济带建设加快推进。长兴岛临港工业区签约项目46个，大阳日酸等32个项目开工建设；实际使用外资5.3亿美元，增长102%。花园口经济区开发建设全面提速，签约项目47个，实际使用外资增长340%。开发区核心产业区、保税区主功能区等8个区域被纳入辽宁沿海经济带重点支持区域，美国固特异、北车工业园等一批重点产业项目相继入驻。经过长期努力争取，旅顺、长海获准对外开放。

　　招商引资和对外贸易实现重大突破。新引进德国博世集团、日本三菱重工等跨国公司投资项目22个，新批1000万美元以上的外资项目102个。外资结构进一步优化，高新技术产业、先进制造业、现代服务业引进外资42亿美元，占总额的81%。外贸进出口双双迈上200亿美元新台阶，分别增长26.5%和20.9%。引进内资410.8亿元，增长21.1%。

（四）改革创新不断深化。国企改革取得实效。完成冰山集团、大耐泵业等股份制改革和大建投等公司制改革。16个厂办集体企业进行了改革试点。建立了国有资本金预算制度。

　　财政、金融等重点领域改革深入推进。建立了长兴岛、花园口和地铁、城市基础设施以及中小企业投融资平台。大连港集团成功发行30亿元公司债券，华锐铸钢等5家企业境内外上市。资本市场直接融资83亿元，是上年的1.5倍。启动了新型农村金融机构和农业保险试点，设立了两家村镇银行和4家小额贷款公司。调整理顺了高新技术园区、花园口经济区和双D港管理体制。推进了事业单位改革和文化体制改革。

　　民营经济发展环境进一步改善。市政府制定了《关于支持民营经济与中小企业持续稳定发展的意见》，为中小企业减免各种税费2000多万元。发行中小企业集合债5.7亿元，为民营企业提供担保贷款80亿元。预计全年民营经济实现增加值2106亿元，增长18%。

　　自主创新能力增强。新增国家级科技企业孵化器3家，培育孵化创新型科技企业1700个。科技创新大厦竣工并投入使用。实施了光电子、数控等8个自主创新专项，启动了3兆瓦风电整机等95个重大技术攻关项目。大连理工大学装备制造研究成果获国家技术发明一等奖。机床集团研制的数控机床关键功能部件和光洋科技开发的双曲轴转台实现批量化生产，风电设备9大关键部件国产化率达85.7%。制造业嵌入式系统公共开发平台正式启动。我市被列为国家节能与新能源汽车示范城市、国家级新材料产业基地和装备制造国际科技合作基地，组建了大连科技创新发展研究中心和大连软件高级经理人学院。新建企业博士后科研工作站5家，引进海外留学人员400人、外埠高端人才1000人。

　　（五）新农村建设扎实推进。农业现代化进程加快。实现农林牧渔及服务业总产值516亿元，增长10%。水产、水果、蔬菜、肉蛋奶产量均创历史新高，粮食生产获得大丰收。新发展设施农业10.8万亩，新建畜牧小区110个。引进农业新品种新技术153个。培训农村实用人才16万人次。金州黄桃获得国家地理标志证明。

　　农村基础设施建设得到加强。完成200个新农村村庄规划编制，全面开展了新农村环境整治工程。投入资金6.8亿元，新建各类重点水源和饮水工程189项，解决了8.2万人饮水安全问题。新建农村公路351公里，解决了3307个自然村出行难问题。

　　农村面貌发生了深刻变化。启动138个生态文明村建设，建设大型沼气工程46处、户用沼气8014个，完成生态移民搬迁930户。实施了5个乡镇生活污水和垃圾管理示范工程。投入2.7亿元用于新农村现代流通服务网络建设。投入1.2 亿元新建234个农村社区服务中心。高标准完成沈大高速公路绿化带建设，实施道路绿化892公里，造林40万亩，植树1亿株。林权改革面积476万亩，占应改面积95.2%。

　　县域经济发展势头强劲。各区市县重点工业园区完成投资419亿元，实现工业增加值312亿元。瓦房店市被确定为“中国轴承之都”。区市县级财政收入增长27.5%，县域经济自主发展能力进一步增强。瓦房店、普兰店、庄河三市全部入选全国县域经济百强县。

　　（六）城市综合服务功能显著提升。城市基础设施建设加快推进。进出市区第二条快速路等交通基础设施建设全面实施，完成了香炉礁立交桥、鲁迅路、五一路等拓宽改造工程，有效缓解了进出市区和部分路段交通拥堵问题。开发区至金州九里快轨建成运营。红沿河核电站一期工程建设加快，哈大客运专线大连段、滨海公路、大伙房水库输水入连、三道沟净水厂改造、大连国际会议中心等工程相继开工建设。供水、供气、供热改造力度加大。

　　生态环境建设取得新进展。实施了西郊国家森林公园、前关城市中央湿地公园、劳动公园改造、滨海路人行木栈道等项目建设。大连冰峪国家地质公园开园。改造了疏港路、迎客路、中山西路和中南路等景观道路，实施了主干道绿化美化工程，新增绿地175万平方米，城市绿化覆盖率达到44%，人均公共绿地面积增至11.6平方米。

　　城市管理水平进一步提高。城市总体规划和土地利用总体规划修编工作全面启动，加强城市管理和执法综合考核，城市管理实现了向微观、向街巷路延伸的精细化管理，城市面貌发生了较大变化。

　　节能减排和环境保护工作扎实推进。夏家河等7座城市污水处理厂建成投产，市区污水处理率达到90%。关停50个造纸企业和23个烟花爆竹企业，淘汰了粘土实心砖生产。华能、庄河等8座燃煤电厂脱硫项目全面启动，其中4座投入运行。全面完成了国家和省下达的减排任务。完成南部海域三期清理任务。建立首个中国绿色碳基金地方专项。全年空气质量创有监测记录以来最好水平。

　　（七）民生质量明显改善。社会保障水平全面提高。就业扶持政策进一步完善，全市实现城镇就业16万人。全面实施了城镇居民基本医疗保险。建立了失业保险金与最低工资标准同步调整机制，企业离退休人员养老金进一步提高。投入社会救助资金2.7亿元，提高了城乡居民最低生活保障标准。慈善事业健康发展。改造农村困难居民危房2000户，新建农村区域性中心敬老院20所，新增城乡养老床位3200张。

　　住房保障体系日益完善。投入廉租住房补贴资金1000万元，市内四区符合条件的家庭实现了应保尽保。80万平方米经济适用住房开工建设。归集住房公积金65.7亿元，发放个人住房政策性贷款29亿元。

　　提高事业单位人员工资收入。全市共筹集安排资金37亿元，提高了中小学教师和事业单位人员的收入水平。

　　食品药品市场整治取得明显成效。启动“食品安全区（市）县建设”工程，及时妥善地解决了三聚氰胺对我市奶蛋产品质量安全的影响。

　　（八）社会事业全面进步。城乡教育协调发展。投资1.3亿元，建设农村标准化学校319所和寄宿学校5所。“北三市”义务教育全面达到了省定“提高普九”标准。免除了城乡义务教育阶段中小学杂费。筹资2亿元，新建市技师学院。

　　公共卫生服务功能不断完善。新传染病医院投入使用。城市社区公共卫生服务体系日趋完善。218万农民参加了新农村合作医疗，人均筹资标准提高到120元，参合率达到98%。爱国卫生工作全面推进，我市成为全国健康示范城市。计划生育率为99.93%，自然增长率为1.25‰。

　　文化体育事业繁荣发展。京剧《风雨杏黄旗》获第五届中国京剧艺术节金奖，电视剧《闯关东》受到观众好评。成功举办第十七届中国金鸡百花电影节。完成人民文化俱乐部改造。旅顺博物馆被评为首批国家一级博物馆，金石滩发现王国被评为国家文化产业示范基地。群众性体育活动深入开展。圆满完成奥运、残奥火炬传递工作。在北京奥运会和残奥会上，大连籍运动员取得了优异成绩。

　　社会管理全面加强。全市第七届社区居委会换届选举圆满结束。群众来信来访总量显著下降，信访秩序明显好转。普法和法律援助、法律服务及人民调解工作取得新成绩。应急管理和安全生产工作进一步加强，社会治安持续稳定， “平安大连”建设取得成效。我市再次荣获全国文明城市，公共文明指数居全国15个副省级城市之首。

　　积极支援抗震救灾。四川汶川特大地震发生后，大连与灾区人民心手相牵，捐送救援物资，派出救援队伍。全市捐赠款物6.3亿元，圆满完成了13256套过渡安置房援建任务，接收救治灾区伤员104 人，妥善安置灾区3000多名大学生在连复课。抗震救灾中，我市的城市精神得到展现和升华。

　　新闻出版、民族、宗教、人防、气象、侨务、对台、防震减灾、档案、残疾人、老龄、双拥共建、民兵和预备役工作取得了新成绩，我市荣获第六轮全国双拥模范城称号。

　　（九）服务型政府建设取得新进展。行政服务体系进一步完善。政府公共服务资源有效整合，构建了行政审批与服务、政务公开、行政投诉为一体的政府行政服务机构，形成了自上而下的四级行政服务体系。全市共建立乡镇、街道便民服务中心154个，村屯、社区代办点2012个。

　　政府管理创新有序推进。完善政务公开、政府信息依申请公开制度，各级行政机关信息公开率达100%。市政府机关全面引入ISO9000质量管理体系，电子政务得到加强。

　　政风行风建设成效明显。深入开展行风热线、在线访谈活动。行政监察工作力度加大，强化了工程项目招投标、国有土地招拍挂、国有产权交易和政府采购的监管，民主评议机关和基层站所工作不断推进。行政投诉网络体系进一步完善，人民群众的合理诉求得到及时有效解决。加强对重点专项资金和重大项目建设资金的跟踪审计力度，提高了财政资金使用效益。

　　认真接受监督。自觉接受人大及其常委会的法律监督、工作监督和政协的民主监督，坚持重大事项向人大报告、向政协通报制度。定期听取人大代表、政协委员和各民主党派、工商联以及无党派人士的意见和建议。816件人大代表建议和721件政协提案全部办结，满意率达95％以上。加强与工会、共青团、妇联等人民团体的联系，召开了市政府与工会联席会议，科学民主决策机制进一步完善。

　　各位代表，我们今天取得的成绩，得益于波澜壮阔的改革开放。30年来，全市上下不断解放思想，坚定不移地推进改革开放的伟大事业。历经改革开放的洗礼，大连经济社会发展取得了令人瞩目的巨大成就。特别是实施东北振兴战略以来，我们加快产业结构高级化、城市功能国际化、经济体制市场化进程，为未来的可持续发展奠定了坚实基础。

　　我们今天取得的成绩，得益于市委的正确领导，得益于市人大及其常委会、市政协和各民主党派的监督与支持，得益于全市人民的同心同德、奋力拼搏。在这里，我代表市政府，向给予政府工作大力支持的人大代表和政协委员，向各民主党派、工商联和无党派人士，向离退休老同志，向中省直各部门和兄弟省市驻连机构，向驻连部队和武警官兵，向全市人民和关心支持大连建设发展的海内外朋友表示衷心的感谢！

　　在充分肯定成绩的同时，我们还必须清醒地看到当前存在的困难和问题：经济持续快速增长的阻力增大，经济结构调整任务还很艰巨，外贸出口困难增多，就业形势日益严峻，节能减排和环境保护压力加大，城乡部分群众生产生活比较困难，交通拥堵的问题没有得到根本解决，一些职能部门行政效率和服务水平还需进一步提高。对此，我们将在今后的工作中努力加以解决。

　　二、2009年形势与任务

　　各位代表，今年是我市经济社会发展面临严峻挑战的一年。当前，国际金融危机仍在继续蔓延，尚未见底，对实体经济的影响正进一步加深，世界范围内的投资、出口和消费全面下滑。今后一个时期，我国经济发展的外部环境更加严峻，经济运行的困难不断增加，经济下行压力继续加大。我市经济外向度较高，受国际金融危机影响突出，特别是出口压力进一步增大，港航物流、装备和船舶制造等重点产业将受到较大冲击。一些中小企业经营困难的局面也将继续加重。这些都会对我市经济发展产生不利影响。对此，我们必须有足够的思想准备。

　　我们也必须看到，我市具备许多抵御金融危机、实现平稳较快发展的有利条件。近年来，市委、市政府坚定不移地推进产业结构战略性调整，加大传统产业改造力度，大力发展高新技术产业和现代服务业，建设了一批重大项目，加快推动产业结构向高级化转型。这种新型的产业结构和多年积累的发展能量，使我市经济的抗风险和可持续增长能力大大增强，这将成为我们加快发展的重要基础。中央扩大内需政策为我市加快重大项目建设、推动产业结构优化升级提供了新的机遇。中央继续加大对东北振兴的支持力度，辽宁沿海经济带开发开放有望上升为国家战略，我市作为东北振兴和对外开放的龙头，将会得到国家和省的更多支持，区位和政策优势必将进一步显现。近几年我市的投资软环境持续改善，生态宜居城市建设水平不断提高，也将使我市聚集更多的先进生产要素，加快振兴发展。只要我们做好充分准备，准确判断，积极应对，从变化的形势中捕捉和把握难得的发展机遇，就一定能够变挑战为动力、化危机为机遇，牢牢掌握发展的主动权，加快推进东北亚重要国际城市和中国北方科学发展示范城市建设。

　　今年市政府工作总的要求是：认真贯彻党的十七大和十七届三中全会精神，坚持以科学发展观统领经济社会发展全局，全面落实保增长、扩内需、调结构的各项政策措施，围绕率先全面振兴，着力调整经济结构和转变发展方式，坚定不移地推进改革开放和创新，加快推进城乡区域协调发展，千方百计改善民生质量，确保经济平稳较快发展、社会和谐稳定。

　　经济社会发展的主要预期目标是：地区生产总值增长14%；万元地区生产总值综合能耗同比降低4%，主要污染物排放总量平均削减5%以上；固定资产投资增长20%；实际使用外资增长15%；地方财政一般预算收入、城镇居民人均可支配收入和农民人均纯收入均增长14%；社会消费品零售总额增长15%；城镇登记失业率控制在3.5%以内。在实际工作中，主要增长指标要力争高于全省水平、高于全国副省级城市平均水平。

　　2009年，市政府工作的主要任务是：

　　（一）努力扩大需求，全力促进增长。把保增长作为全年经济工作的首要任务，坚持扩大内需与扩大外需并举、需求拉动与供给推动并重，确保经济持续增长。

　　加大投资力度。落实一批对我市今后长远发展和产业结构调整发挥重要作用的重大项目，固定资产投资超过3000亿元。开工建设城市地铁一、二号线和机场三期扩建工程，启动新机场建设前期工作，开工大连至旅顺、推进金州至普兰店及金渤海岸的轨道交通建设。实施“五大城际交通工程”，推进哈大客运专线及南关岭综合交通枢纽建设，启动京大客运专线、庄盖高速公路建设，完成辽宁滨海公路大连段建设。加快红沿河核电站建设，开工一批热电厂建设项目。加快大伙房输水工程建设。结合旧城区改造和新区建设，加大房地产投资力度。

　　扩大消费需求。要提高消费需求对全市经济增长的贡献率。千方百计增加城乡居民收入，不断提高人民群众的消费能力，促进消费结构升级。继续加大政策引导，鼓励市民扩大住房消费，并以此带动相关联消费。把扩大消费与现代服务业发展紧密结合起来，积极开展城市营销，加大旅游推介力度，积极申办国际国内重大会议和展销活动，将域外购买力转化为我市的消费需求。加大全市商业设施改造力度，积极开发文化娱乐、体育健身等热点消费。全面启动农村消费市场，扩大农村消费。积极引导大商集团等有实力的大型商贸企业开拓农村市场，启动6个农村中心城镇商业建设，新建和改造一批农家店和农村商品配送中心，加快推进农机、家电下乡工作。创造有利条件，拉动我市地方产品的消费需求。规划建设2个大型农副产品集散中心，搞活农产品流通。

　　全力扩大出口。积极为扩大出口创造良好的政策环境和服务环境。实施出口监测制度，对出口100强企业实行全程跟踪服务，及时解决进出口遇到的各类问题。综合运用出口退税、中小企业开拓国际市场专项资金等政策措施，扶持企业扩大出口。实施出口多元化战略，以增量弥补减量，确保对外贸易稳定增长。

　　发挥企业作用。企业是经济之源，保企业就是保增长、保就业、保稳定。全力支持新上项目的企业尽快竣工投产，形成新的生产能力。支持市场销路广、经营效益好、投资能力强的企业进一步扩大生产规模，有条件的企业要通过国内外并购的方式增强国际竞争力，扩大市场占有率。要引导企业适应市场变化，积极调整市场结构和产品结构，加大技术改造力度，强化管理，降低成本，提高市场竞争力。要积极支持中直企业做强做大，进一步推动市属企业的改革重组，提高其竞争力。创造宽松环境，促进中小企业的稳定发展。要建立困难企业突发事件协调应对机制，政府部门与金融机构和审判机关相互协调，统筹解决突发事件，防止企业非正常倒闭和大规模裁员。

　　（二）加快结构调整，提升产业竞争力和可持续发展能力。按照国家新的宏观调控政策和新产业振兴规划要求，着眼扩大内需形成的巨大市场需求，促进工业结构调整，推进产业体系向高端化发展，提高我市产业的国际竞争力和可持续发展能力。

　　打造一批世界级产业基地。依托“两区一带”和沿海各重点区域，推进一批重大项目建设。大力发展国家重点支持的高效清洁能源和节能、环保重大装备等新兴产业，推进重工·起重风电装备产业化，加快瓦房店风电设备制造基地建设，支持中国一重集团建设国内最大的核岛装备生产基地，把大连打造成为世界级风电和核电设备生产基地。全力推进节能与新能源汽车示范项目建设，规划建设新能源汽车园区，支持一汽集团在大连建设混合动力和电动客车生产基地，促成电动汽车总成项目，通过招商引资尽快形成完整的新能源汽车产业集群。大力发展汽车零部件产业，加快美国固特异轮胎、德国大众自动变速箱、美国博格华纳变速箱模块等项目建设，争取在整车项目上有所突破。加快北车集团旅顺机车产业园建设，推进大功率交流传动电力机车和城市轨道车辆项目，形成世界级的轨道交通装备基地。推进英特尔芯片工厂和大连半导体产业园建设，吸引更多的配套企业进驻开发区，打造世界级集成电路产业基地和半导体产业集群。

　　推进船舶重工、STX、中远、中集、万邦等造修船和海洋工程项目以及重工·起重船用曲轴扩能、船柴扩建等船舶配套项目建设，加快建设中国最大的造船基地。推进大孤山半岛石化产业园区建设，加快推进长兴岛中石油炼化和双岛湾石化项目，拉长石化产业链条，形成产业集聚区，提高石化产业的核心竞争力。推进国际油品储运基地建设。

　　大力发展现代服务业。加快旅顺南路软件产业带和大东沟服务外包基地建设，努力开拓国际市场，积极引进外包业务，全力推进自主创新，大力发展具有自主知识产权的嵌入式软件和应用软件，做强做大软件企业，建设全球软件和服务外包新领军城市。拓展大窑湾保税港区保税加工、国际中转等业务，积极引进国际知名物流企业，实现航运、港口和现代物流一体化发展。加快香炉礁、空港、羊头洼等七大物流园建设。提升中山广场金融商务区功能，星海湾国际金融中心要全面投入使用。支持大连银行开设沈阳等分行，争取大商所上市期货新品种。加快世茂嘉年华等重大旅游项目建设，推进金渤海岸现代服务业发展区和长山群岛国际旅游度假区建设。巩固日韩旅游市场，拓展俄罗斯、中东和台湾地区等旅游市场，大力开发商务会展、休闲度假等高端旅游产品。加快东港商务区建设，推动新世界、时代广场、恒隆广场等高端商业区建设，加快天津街、西安路、长春路、香炉礁、中华路商业区、街升级改造。

　　（三）加大改革创新力度，增强经济社会发展的动力与活力。推动重点领域和关键环节改革向纵深发展，努力破除制约发展的体制机制障碍；加快创新型城市建设步伐，不断增强自主创新能力，为保增长、促发展注入新的活力和持久动力。

　　继续深化国有企业改革。推进国有大型企业股份制、公司制改革，加强国有企业现代企业制度建设和重组兼并工作，增强国有企业实力，通过重组做强做大我市装备制造业。完善国有资产监督管理体系，实现国有资产保值增值。

　　推进财政、金融等重点领域改革。认真贯彻中央积极的财政政策和适度宽松的货币政策，加快投融资体制改革，推进投融资平台建设，建立健全政府投资管理体制和新型投资体制，积极推动企业在境内外上市。完善农村金融服务体系，深化农信社改革，做好村镇银行等新型农村金融机构组建工作，推进小额贷款公司试点工作，扩大农业保险试点范围。继续推进甘井子区和普兰店市综合配套改革试点，加快城市化进程。

　　全面实施创新驱动战略。重点实施新能源、生物技术等10个重大专项，组建和启动风电、轴承等8个技术创新联盟，创办数控技术研究院、光电子研发中心等10个具有国际水平的研发机构，启动百佳创新型企业示范工程，引导和推进创新资源向产业高端集聚。着力突破制约产业转型升级的重大关键技术，在高端通用芯片、超大规模集成电路、高档数控机床等应用技术领域取得进展。开发一批自主创新战略产品，培育一批高成长性、创新型企业和企业集团，加快形成战略性高技术产业集群。加快中科院大连创新园建设。推进开放式创新，实施海内外自主创新企业及核心专利技术并购计划，吸引发达国家和地区研发、设计、工程服务和创业投资机构入驻大连。

　　（四）继续推进对外开放，全面提高开放型经济水平。准确把握当前国际经济发展走势，紧紧抓住国家支持辽宁沿海经济带开发开放的历史机遇，在更广领域、更大规模和更高水平上推进对外开放。

　　进一步拓展对外开放领域。开放先导区要创造新的开放优势，实现新的跨越。开发区要以小窑湾开发为契机，着力提升整体服务功能；以双D港为载体，打造世界级集成电路基地和国家级生物产业基地；依托大窑湾港打造汽车整车及零部件产业和新能源汽车基地。保税区要紧紧抓住国家特殊监管区域整合发展的重要机遇，实现政策再突破、功能再提升、辐射再扩大。加快金州二十里堡综合配套区基础设施建设，实施区域空间和引进内外资倍增计划。高新园区要以空间和体制调整为动力，高水平规划建设旅顺南路软件产业带，打造世界一流的软件和服务外包基地，成为建设全球软件和服务外包新领军城市的核心区。长兴岛临港工业区要以建设“辽宁特别经济试验区”为载体，大力引进国家重点企业和国际大公司重大项目，全力打造世界级产业聚集区。花园口经济区要以建设国家新材料产业基地核心区为依托，加快形成新材料产业的聚集效应，打造国内一流的新材料产业基地。旅顺和长海要以创新思维、国际化标准，科学制定发展规划，创造对外开放的崭新格局。“一岛十区”的其它重点区域要选准定位，有针对性进行招商引资，发展特色园区，培育产业集群，创造沿海开发开放的新优势。市内各区要围绕“总部经济”加大招商引资力度，完善市中心的国际化功能。推进北黄海开发开放，做大做强庄河实力，形成新的对外开放热点。

　　全力推进投资合作。要充分利用当前发达国家经济衰退所引发的产业结构新一轮调整的机遇，加大装备制造业、高新技术产业和现代服务业的招商引资力度，在汽车整车、新能源汽车、集成电路、服务外包等重大项目上有新突破，进一步优化我市产业结构，提高产业的国际竞争力。要加大城市基础设施和房地产利用外资力度，全力推进东港区开发的招商引资工作。要加快实施走出去战略，积极支持企业到国内外收购一批科技企业和研发机构，充分发挥大连日本软件园的窗口作用，为软件企业跨国兼并和引进人才服务。

　　着力提升国际航运中心功能。继续推进大连港与省内各港口的战略合作，整合北良、辽渔及区市县港口资源，建立起以大连港为中心，功能互补、辐射力强的航运中心组合港。完善环渤海内支线和连接东北腹地的海铁联运网络。加快构建以保税港区为核心、以腹地干港和保税物流中心为节点的东北保税物流网络。加快大窑湾集装箱码头和大窑湾、长兴岛核心港区的公用基础设施建设，推进新30万吨原油码头、庄河石城深水港和旅顺新港区海上滚装运输枢纽工程建设，完成金州杏树国家级中心渔港、10万吨油码头及长海陆岛码头建设，启动长兴岛30万吨原油码头建设。加快长兴岛、大连湾疏港高速公路和长山群岛陆岛交通建设。继续增加空港国际航线，加快空港物流园建设。推进大窑湾三期集装箱码头口岸开放申报、长兴岛港口岸和大孤山工业园口岸开放工作，完成旅顺新港和庄河港的口岸开放验收工作。

　　举全市之力筹办好今年的夏季达沃斯论坛，并积极争取其永久落户大连，成为大连走向国际化的新平台。

　　（五）推进新农村建设，加快城乡一体化进程。把建设社会主义新农村作为战略任务，不断加大对“三农”的支持力度，开创城市带动农村、沿海带动全域、工业化带动城镇化的发展新局面，不断壮大县域经济实力。

　　加快农村工业化和城镇化进程。推进县域工业园区建设，加大内外资项目引进力度，引导资金、技术、人才等生产要素快速向园区聚集，使工业园区尽快成为县域经济发展的先导区和重要支柱。提升城市化水平，将“南三区”纳入城市发展总体规划，推进郊区向城区转变、农村向社区转变。在“北三市”分层次、有重点地建设一批工业型、商贸型、旅游型等不同特色的小城镇，形成城镇化和新农村建设互促共进机制。推进农村社区建设，完成农村社区服务中心建设工程，覆盖面达到60%。加快发展县域服务业，以中心镇商业建设为龙头，带动农村服务业发展。通过旅游业发展促进农民增收。

　　积极发展现代农业。大力发展特色农业和优势产业基地建设，制定“一村一品”、“一乡一业”发展规划，加快推进优势特色农产品区域化、规模化、专业化、标准化生产，新发展设施农业10万亩。加强高附加值的现代海洋渔业基地建设，培育壮大一批起点高、规模大、品牌好、带动力强的农业龙头企业。加速推进农产品标准化生产，建立严格的农产品质量安全监督检验体系，确保农产品质量安全。积极培育创建国家、省、市级名优农产品品牌。扩大农业对外开放，加快农业国际化进程。创建国家级农业科技成果转化中心。建立农业技术推广、动植物疫病防控、农产品质量监管体系。

　　加强农业和农村基础设施建设。积极发展节约、循环、生态型农业。抓好农田水利基本建设，搞好生态河治理，完成病险水库除险加固工程。大力推进生态文明村、荒山造林补植等“八大林业工程”。加快新农村示范村建设，积极推进农村安全自来水、垃圾和污水处理、村镇路网、沼气工程等新农村建设“十大工程”。大力发展农村公共事业，努力缩小城乡差距。

　　积极稳妥地推进农村改革。稳定和完善农村基本经营制度，健全严格规范的农村土地管理制度，鼓励多种形式土地流转，积极推进规模化经营。搞好林权制度改革，成立县区土地、林权交易中心。

　　积极扩大农民就业。加强对农民转移就业和返乡创业的服务，增加培训投入，扩大培训规模，广开就业门路；引导农民向二、三产业和城镇有序转移就业，拓宽就业渠道和增收空间，确保农民持续稳定增收。　　 （六）加强城市建设管理，提高生态环境水平。全面完成城市总体规划和土地利用总体规划修编，加强城市基础设施和生态环境建设，创新城市管理方式，促进城市综合服务功能全面提升，提高城市宜居水平。

　　大力推进以城市路桥为重点的交通基础设施建设。完成东联路、东北路南段和迎客广场立交桥等建设工程，拓宽改造胜利路西段等城市道路。调整优化公交线网布局，努力提高道路通行效率，构建畅通、便捷的公共交通网络，使交通拥堵问题得到初步解决。

　　进一步完善城市功能。推进东港区开发改造，加快国际会议中心建设。在市内四区、开发区、高新园区实施“开发改造一片旧城区、新建一座停车场、改造建设一个菜市场、新建一处市民健身场所、改造提升一条路街环境”的“五个一工程”；在其它区（市）县实施“开发改造一片旧城区、改造建设一个集贸市场、改造提升一条路街环境”的“三个一工程”，不断完善城市功能，提高市民的生活质量。

　　加大生态环境建设力度。全面启动森林城市创建工作，大力实施以植树为重点的城市园林绿化建设工程。改造植物园和西山览胜景区，建设滨海路绿之梦广场、白云雁水健身公园，推进西郊、英歌石和大黑山森林公园建设，加快河道生态工程建设。推广城市景观亮化工程和节能照明。以迎接夏季达沃斯论坛为契机，加大城市净化、绿化和美化力度。

　　深入推进节能减排。明确责任、强化监督，严禁高耗能、高污染项目进入。加快淘汰落后产能，大力推广节能示范工程。完成大钢、大化等企业搬迁改造。加快燃煤电厂脱硫改造，启动燃煤工业锅炉节能减排项目，加大拆炉并网工作力度，加强机动车污染防治，发展碳汇林业，提高城市空气质量。实施雨污分流的城市排水管网改造工程，加快推进营城子等8座污水处理厂建设和改造，启动虎滩新区、头道沟污水处理厂建设工程。开工建设中心城区生活垃圾转运及垃圾焚烧厂，构建城市新型生活垃圾除运体系。

　　提升城市现代化管理水平。继续加大城市综合整治工作力度，全面开展城市管理考核工作。搭建城市管理信息化平台，逐步导入数字化城市管理新模式，增强信息基础设施的综合服务能力。

　　（七）发展各项社会事业，促进和谐大连建设。坚持经济社会统筹协调发展，推动社会事业全面进步，不断满足广大市民日益增长的物质文化需求。

　　促进义务教育均衡发展。加强教师队伍建设，强化素质教育。整合职业教育资源，启动涉农专业农村学生免费职业教育，新建1所高标准寄宿制中等职业学校，逐步实施农村中等职业学校免费教育。实施中小学免费体检计划和健康饮水工程，规范农村中小学校车管理和运行机制。支持在连高校加快发展。

　　继续完善公共卫生体系。加强社区卫生服务体系建设，完善“六位一体”的社区卫生服务功能。提高新农合筹资标准，增强保障能力。完善市区急救网络，推进市疾病预防控制中心和市心理医学中心建设，改造市儿童医院。深化爱国卫生工作，继续开展健康城市建设，着力提高全民健康素质。落实计划生育奖励政策，实现计划生育优质服务全覆盖。

　　大力发展文化和体育事业。加强公共文化体系建设，完成现代博物馆改陈工程，完成汉墓博物馆和乡镇文化站建设，规划建设特色文化街区。创演一批地域文化色彩浓厚、群众喜闻乐见的文化艺术作品，开展丰富多彩的群众文化活动。支持大连京剧院等单位争创全国名院（团）工作。积极稳妥地推进文化体制改革。广泛开展全民健身运动，努力提高竞技体育水平。举办好大连市第十一届运动会、第二十三届大连国际马拉松赛及第七届大连国际徒步大会。加快市体育中心、新市民健身中心等公共体育设施建设。

　　继续做好新闻出版、广播影视、民族、宗教、侨务、对台、档案、人防、气象、防震减灾、双拥共建、民兵和预备役等工作。

　　（八）深入实施民生工程，努力提高人民群众生活水平。推进“富民工程”的实施和市政府为民办实事的落实，切实解决人民群众最关心、最直接和最现实的利益问题，不断提高人民群众的生活水平。

　　努力扩大就业和再就业。实施更加积极的就业政策，发挥重大投资项目带动就业的作用，千方百计增加就业岗位。全面贯彻《就业促进法》，进一步完善就业扶持政策，大力推动创业就业。建成市技师学院和实训基地，加强高技能人才队伍建设，改善劳动力素质结构，推动素质就业。发挥就业援助机制作用，帮助高校毕业生和困难人群实现就业。研究制定进一步稳定和促进就业措施，加强失业调控，严格控制和规范企业裁员行为。全市实现城镇就业12万人，保持就业局势基本稳定。

　　健全覆盖城乡的社会保障体系。进一步扩大社会保险覆盖面，完善城镇企业职工养老保险制度，将农民工纳入养老保障范围，探索建立新型农村养老保险制度。做好被征地农民养老保障工作，提高城乡居民最低生活保障、企业退休人员养老金和城镇职工基本医疗保险待遇。加强劳动关系协调体系建设，深入实施劳动关系和谐工程，依法维护劳动者合法权益，促进劳动关系和谐稳定。

　　完善城乡社会救助体系。建立城乡最低生活保障标准动态调整机制，进一步提高保障标准。完善高中困难学生助学体系建设，为8000名家庭困难的高中生年均发放助学金1000元。全面完成农村区域性中心敬老院建设和农村困难居民危房改建五年计划任务。推进城市养老服务社会化和居家养老服务工作，加强老年服务设施及活动设施建设。为1000名残疾人实施托养，创建全国残疾人工作示范城市。继续实施生态移民工程。支持慈善事业发展。组织好对口帮扶工作，完成对口支援任务。

　　加快保障性住房体系建设。进一步提高住房公积金覆盖面和政策性贷款惠及面，拉动房地产市场有效需求。全面推进城市新居工程，安排经济适用房8000套，扩大廉租住房保障范围。强化对房地产市场的引导和调控，加大对房地产交易违法违规行为的整治力度，促进房地产市场健康平稳发展。

　　加强市场监管和价格调控。深入开展整顿和规范市场秩序专项行动，维护市场经济秩序。实施城市“早餐工程”和社区“菜篮子”便利工程，逐步建设社区标准化菜市场。

　　（九）强化社会管理，全力维护社会稳定。在当前经济发展遇到困难的情况下，加强和改善社会管理，做好维护社会稳定工作尤为重要。

　　巩固和发展全国文明城市创建成果。深入开展文明单位、文明行业、文明村镇等群众性精神文明创建活动，不断提高市民文明素质和城市文明程度。大力加强思想道德建设，深入普及公民道德基本规范，在全社会形成讲文明、树新风、学先进的良好风尚。积极构建学校、家庭、社会“三位一体”的未成年人思想道德教育网络，为青少年健康成长营造良好的社会环境。加强社团组织的建设与管理，促进其健康发展。开展多种形式的双拥共建活动，再创全国双拥模范城。

　　加强民主法制建设。深入推进“五五”普法规划的实施，做好法律服务和法律援助工作，深化“法律进乡村”等“六进”活动，加强人民调解工作和城乡基层民主建设，规范社区社会组织的发展，全面推进和谐社区建设。

　　强化应急管理和安全生产工作。完善应急预案和应急管理标准体系建设，健全应急专家和救援队伍，增强应急管理和救援能力。全面加强安全生产工作，建立安全综合决策机制，实施城市安全评价工程。继续深入开展隐患排查治理和安全专项整治，坚决遏制重特大事故发生。

　　高度重视食品药品安全。完善食品药品安全责任体系，强化检验检测能力和执法队伍建设，深入整顿和规范市场秩序，确保群众饮食用药的安全，全面推进“食品安全城市”建设。

　　切实维护社会稳定。强化社会治安管理，严厉打击违法犯罪活动，争创新一轮全省综合治理工作先进城市。进一步深化大接访活动，加大信访调处力度，努力解决信访突出问题。加强人民内部矛盾纠纷的排查调解，促进社会和谐稳定。

　　自觉接受人大及其常委会的法律监督、工作监督和政协的民主监督。继续坚持重大事项向人大报告、向政协通报制度，认真做好人大代表建议、政协提案办理工作。

　　各位代表，今年的各项任务异常繁重艰巨，能否在严峻的形势下，保持经济社会持续健康发展，这对我们来说是一个巨大的考验。我们要增强紧迫感和责任感，科学判断形势，积极应对困难，采取有效措施，圆满完成全年各项任务，向全市人民交上一份满意的答卷。

　　（一）保持奋发向上的精神状态。面对当前严峻复杂的形势，保持良好的精神面貌至关重要。要把我们的思想统一到中央对经济形势的分析和判断上来，统一到中央的决策和部署上来，统一到科学发展的要求上来，坚定发展信心不动摇，咬住发展目标不放松。要看到，当前的严峻形势带有普遍性，谁保持了高昂的政治热情、坚韧不拔的奋斗精神、坚定必胜的信心和决心，谁就会战胜困难，赢得主动。还要看到，当前是挑战与机遇并存，挑战中蕴含着机遇，只要我们以积极的姿态、创新的思维，科学谋划，趋利避害，就一定能够化危机为机遇，实现新一轮更好更快发展。多年实践证明，一些城市就是在别人认为是困难的环境中，靠抢抓机遇，逆势而上，实现成功崛起的。各级政府及工作人员要牢固树立为我们城市长远发展和繁荣负责的意识，发扬迎难而上的拼搏精神、抢抓机遇的进取精神、求真务实的科学态度，破解发展难题，攻克发展难关，打赢保增长的攻坚战。

　　（二）加大政策扶持力度。在经济出现下行压力时，政府实施积极的调控政策是非常重要的。要认真贯彻落实中央扩大内需、促进经济平稳较快发展的各项政策。结合我市具体情况，针对出口受阻、房地产销售下滑、企业经营困难、失业压力加大等问题，及时出台支持企业发展的有关政策措施，运用财政、税收、金融、工商和劳动保障等杠杆，以扩大需求、减轻企业负担为主要目的，加大政府对经济增长的拉动力度。市政府刚刚出台了《大连市支持中小企业稳定健康发展的若干政策措施》、《关于促进房地产市场健康稳定发展的指导意见》、《关于进一步促进软件和服务外包产业发展的若干规定》，即将出台《关于进一步稳定和促进就业工作的意见》，这些政策涉及政府直接给予补贴或减轻企业负担的资金共30多亿元。各级政府和政府各部门要认真贯彻落实，使每一条政策措施尽快见效。今后一段时间，市政府将根据经济运行出现的新情况、新问题，及时出台相应政策和措施，加大对经济的调节力度。

　　（三）全力做好要素保障工作。要实现保增长、扩内需、调结构的任务，必须确保要素供应。资金供应的保障是今年经济工作的重中之重。要积极争取国家的支持，争取更多的国债资金和专项补助资金。建立产业基金，解决产业发展中的资金瓶颈。推进投融资平台建设，多渠道解决中小企业融资难问题，综合运用各类融资工具，引导金融资金、民间资金和社会资金对中小企业的支持，今年银行新增贷款要超过1000亿元。积极争取发行地方债券，增强政府财力投资能力。发挥财政资金的示范引导作用，促进投资主体多元化。积极发展多层次资本市场，大力推进企业在境内外上市。加大电力、水利等基础设施建设，做好煤电油运的调度，保证企业生产需求。着力培养和引进高层次人才和高技能人才，为经济社会发展提供坚实保证。

　　（四）进一步提高行政效能。今年能否圆满完成各项经济社会发展任务，是对政府行政能力的巨大挑战。进一步提高行政效能，关系到中央宏观决策能否得到落实，关系到能否创造良好的投资环境，增强我市对资本、技术、人才的吸纳能力和企业的竞争力，关系到老百姓对政府的信任。各级干部和公务员要进一步转变作风、狠抓工作落实，把全年的各项目标任务落实到每天的工作日程，做到事事有着落，件件有回音。要提高处理复杂问题的能力，在重大项目征地、征海、动迁等工作中，既要保持社会稳定，又要做到高效及时。要加快行政管理体制改革，进一步减少行政审批事项，优化工作流程，缩短审批时限，严禁推诿扯皮。继续推行ISO9000质量管理，进一步优化政府服务，帮助企业和市民解决遇到的困难，坚决反对不作为现象。要厉行节约，严格控制一般性支出，大力压缩机关公用经费。严格控制出国考察，停止购买办公车辆。坚决制止铺张浪费、奢靡之风。要强化考核督查。把保增长的各项政策措施的落实情况作为绩效考核的重点，加大督查力度。强化对领导干部的经济责任审计和政府重大项目的跟踪审计工作。加强执法监察、廉政监察和效能监察，依法严肃查处违法违规行为，建设风清气正、富有活力的服务型政府。

　　各位代表，今年我们将迎来新中国成立60周年。我们的城市将站到一个新的历史起点上。肩负时代赋予的使命和600万大连人民的重托，我们不能有半点懈怠，唯有积极进取和不懈奋斗。让我们在党中央、国务院，省委、省政府和市委的坚强领导下，高举中国特色社会主义伟大旗帜，深入贯彻落实科学发展观，万众一心，克难奋进，为实现率先全面振兴的目标而努力奋斗！
